# Comparative Analysis of Emergency Planning Zone and Control Room Habitability for Potential Nuclear Reactor Deployment in Ghana

**DOI:** 10.3390/ijerph191811184

**Published:** 2022-09-06

**Authors:** Prah Christina, Juyoul Kim

**Affiliations:** Department of NPP Engineering, KEPCO International Nuclear Graduate School, 658-91 Haemaji-ro, Seosaeng-myeon, Ulju-gun, Ulsan 45014, Korea

**Keywords:** RADTRAD, RASCAL, fuel handling accident, rod ejection accident, long-term station blackout, exclusion area boundary, low population zone, control room

## Abstract

Following the recent surge in harnessing clean energy sources to fast-track carbon neutrality, renewable and nuclear energies have been the best-rated sources of clean energy. Even though renewable energy presents an almost insignificant risk to public health and the environment, they are insufficient to support the growing demand for the high energy required for industrialization. Despite the competitive potential of nuclear energy to meet these demands, public concerns about its safety have significantly hindered its mass deployment in developing countries. Therefore, one of the primary considerations in commissioning a nuclear power plant is the establishment of emergency planning zones based on the reactor type and other siting criteria. Based on Ghana’s reactor type assessment (RTA), four reactor designs were considered in this study which are APR1400, HPR1000, VVER1200, and Nuscale Power Module. Using the NRC’s SNAP/RADTRAD and RASCAL codes, this research sought to investigate radionuclide doses released at the Exclusion Area Boundary (EAB), Low Population Zone (LPZ), Control room (CR), and the 16 km recommended public safe zone during Fuel handling Accidents (FHA), Rod Ejection Accident (REA), and Long-Term Station Blackout (LTSBO). The results revealed that reactors’ power contributed to the source term activities and offsite consequences during REA and LTSBO, while FHA was predominantly affected by the number of fuel assemblies and a fraction of damaged fuel. Additionally, the accidents considered in this study followed a similar trend of impact in decreasing order of reactor power and the number of fuel assemblies; APR1400 < VVER1200 < HPR1000 < Nuscale. Nevertheless, all the doses were within regulatory limits.

## 1. Introduction

Ghana is located in the sub-Saharan part of Africa, with a population of about 31 million [1]. In 2020, the country’s GDP increased by 7.62%, making it $72.35 billion [2]. With the tremendous increase in industrialization, Ghana’s energy sector has been exponentially impacted over the past ten years. Its energy mix includes hydroelectricity, thermal, natural gas, and solar. The total capacity for existing power plants is 4132 MW, with hydro taking up 38%, 61% thermal, and less than 1% solar energy [3]. Ghana’s nuclear vision evolved as early as 1964 post-independence. As part of the country’s commitment to the peaceful use of nuclear energy, Ghana deployed the 30 kW GHARR-1 research reactor from China, which has been in operation since 1995 [4]. Therefore, it has become expedient for the government to begin deploying commercial reactors to satisfy the uprising demand for electricity. Following Ghana’s long-standing history of using nuclear materials since 1995, it has now taken steps to deploy nuclear power technology. Presently, four pressurized water reactors are under consideration. The Advanced Power Reactor (APR1400) is an enhancement in the OPR1000 PWR designed by KEPCO E&C under the Korean government-led G-7 project. It is designed with robust safety features to overcome some natural disasters and plane crashes [5]. Its extended operational lifetime from 40 to 60 years makes it economically competitive; however, the initial investment cost required for deployment has been a major setback for many developing countries considering nuclear technology [6,7]. Also under review is the VVER1200 Russian technology with a net capacity of 1114 MWe, which incorporates Gidropress-design PWR as an advancement of the VVER-1000. The integration of high-level automation in VVER1200 reduces operator interaction by 25–30% compared to VVER-1000 [8]. The Hua-long Pressurized Reactor (HPR1000) is an evolution of existing PWRs infused with passive and active safety systems [9]. Like most current generation III reactors, HPR1000 provides preventive and mitigatory measures for severe accidents due to its enhanced protection against external events and improved emergency response capacity [10]. Finally, the American Nuscale Power Module (NPM) has been the most recent sensation in the energy industry. The design is suitable for growing industries and affordable for middle-income communities. NPM is a 12-module small modular PWR generating 77 Mwe electric per module [11]. Alternatively, depending on the energy demand, the modules may be downsized to six or four [12]. Nuscale design eliminates major features in conventional reactors, such as the direct and alternative currents, due to its passive safety system allowing automatic shutdown without operator intervention [13]. The NMP design promises less severe accident tendencies, reduced radiation emission, and reduced spent fuel waste by an appreciable percentage compared to large reactors [14]. Ghana’s technical basis for considering PWRs is due to the constant availability of water to serve as coolant and moderator for safe reactor operation [15]. The numerous studies conducted about Ghana’s future nuclear energy production have focused much attention on the feasibility of conventional reactors. However, small modular reactors (SMRs), being the first of a kind, are yet to receive country-specific attention on their relevance. Considering Ghana’s economy and its 5300 MW grid capacity, experts say SMRs are an ideal venture in the nuclear industry. SMRs can be deployed to remote communities with less accessibility to electricity as well as industrial support activities that impact economic development. In addition, the coastal availability of the country will make the transportation and operation of small modular reactors easier. Therefore, SMRs can be the breakthrough for sustainable energy in Ghana. The significance of this study is to generate an analysis that will influence the evidence-based selection of nuclear power reactors considering the safety of the public and environment, having Ghana’s Reactor Type Assessment (RTA) in perspective.

Nuclear power has sustained a long-standing history of providing the maximum and affordable electricity supply required for growing industries amidst past safety incidences. However, the mass deployment and commercialization of nuclear power plants in developing countries face some setbacks due to the obvious safety concerns of every first-of-a-kind (FOAK) technology. Notably, the historical tragic events associated with the nuclear industry, such as the TMI, Chernobyl, and Fukushima, raise public and regulatory skepticism regarding its safety and security [16]. However, lessons learned from historical nuclear events have influenced improved safety features embedded in recent power plants and their operational guidelines to protect public health and environmental integrity. Inherently, it is established that the backbone of every risk-associated operation is its emergency preparedness and planning to protect the environment and the public. Therefore, before any nuclear facility is installed, the IAEA recommends nineteen (19) critical issues to be addressed, including emergency planning [17]. In a nuclear emergency, the Emergency Planning Zone (EPZ) is the area surrounding the nuclear power station where additional considerations and management techniques are prepared and practiced. EPZ criteria are tackled before deployment using parameters established from evidence-based research on related reactors, site climatic conditions, and other environmental characteristics [18]. The general EPZ size for power reactors is 16 km for the plume exposure pathway and 80 km for the ingestion exposure pathway, according to 10 CFR 50.33(g) and 50.47(c)(2) [18]. On the other hand, the EPZ size for reactors with power output less than 250 MWth can be determined on a case-by-case basis. Internationally established regulatory criteria are critical for reducing the radiological impact on public health and the environment. According to the US Nuclear Regulatory Commission (NRC), emergency planning zones are divided into plume exposure and ingestion exposure pathways. EPZs vary in size due to site-specific factors such as terrain, weather, and demographic data. The first EPZ is a plume exposure pathway that circles the reactor site up to 16 km from the power station. In this field, protective action plans are developed to prevent or limit doses from potential exposures through the inhalation of radioactive particles. These actions include shielding, evacuation, and administering potassium iodide pills. The second EPZ is a 50-mile-radius ingestion exposure pathway surrounding the nuclear site. This area’s protective action plans are meant to avoid or limit the doses received from ingesting or drinking radioactive materials. One of these measures is prohibiting contaminated food and drink [18].

Control Room Habitability (CRH) systems allow control room personnel to remain safe while mitigating radiological release consequences through safe plant operations in case of accidents. The control room’s sizes and volumes vary with the reactor type. However, the maximum occupancy for most standard reactors is ten operators [19]. Control room operators are protected by CRH equipment from smoke outside the control room and gas leaks. In addition, secure facilities equipped with requisite materials are provided for operators to allow a prolonged stay in the control room envelope (CRE). There are already regulatory thresholds within which the control room is considered safe. According to the Code of Federal Regulations, 10 CFR 50.34, control room doses should not exceed 0.05 Sv/50 mSv/5 rem to ensure habitability within 72 h of accident progression [20]. The Exclusion Area Boundary (EAB) is the closest distance surrounding a nuclear power plant where the licensee has the authority to determine all activities, including exclusion or removal of personnel and properties from the area. A standard nuclear reactor’s exclusion area boundary ranges from 800–6000 m. Notwithstanding, with current reactor safety systems in place, EAB may even be lower, especially for SMRs, which will be determined on a case-by-case basis [21]. Low Population Zone (LPZ), however, refers to the area immediately around the exclusion zone that contains residents whose total number and density are such that suitable protective measures could be implemented on their behalf in the event of a catastrophic accident. Although the population density for this zone is unspecified, residents in this area should not exceed 25,000 people, which is the criteria for a safe zone beyond 16 km from the power station [17]. In addition, the NRC Regulatory Guide 1.195 and 10 CFR 100 recommend that a person located within the exclusion area boundary (EAB) for two hours from the start of a severe accident may not receive an estimated whole-body dose exceeding 25 rem/250 mSv or a thyroid dose exceeding 300 rem/3000 mSv [21]. The above criteria are the same for the low population zone for an individual residing within 30 days of accident progression [22]. Adopting the SNAP/RADTRAD and RASCAL codes to simulate REA, FHA, and LTSBO, this project seeks to assess the maximum doses at the emergency planning zone and control room of the four reactors considered in Ghana’s Reactor Type Assessment (RTA), which are the APR1400, VVER1200, HPR1000, and Nuscale Power model. The REA and FHA simulation output will estimate the doses at the EAB, LPZ, and control room. The thyroid and whole-body doses are also calculated based on the iodine and noble gases released into the environment. RASCAL simulation results will be used to evaluate the doses at the recommended safe distance of 16 km from the power plant. Finally, the contributing factors affecting the resulting dose estimation will be critically analyzed.

## 2. Materials and Methods

The method used in this study was based on the SNAP/RADTRAD manual and the APR1400 Design Control Document Chapter 15 [23]. The RADTRAD analysis mathematical models used in calculating the EAB, LPZ, and CR were adopted from the FHA report conducted for Exelon Nuclear and Grand Gulf Nuclear Station [24,25]. The severe accident analysis conducted using RASCAL was purely based on the code’s manual. Table 1 displays some reactor-specific parameters used for the RADTRAD and RASCAL simulations.

### 2.1. SNAP/RADTRAD Model

The Symbolic Nuclear Analysis Package/Radioactive Transport, Removal, and Dose Estimation (SNAP/RADTRAD) code developed by the U.S NRC has been used to calculate radionuclide doses discharged to the exclusion area boundary (EAB), the low population zone (LPZ), and the control room during a postulated design basis accident. In addition, for various loss-of-coolant accidents (LOCAs) and non-LOCA incidents, the code is critical in validating license compliance with nuclear power plant regulatory siting criteria and control room dosage thresholds. Given this, the SNAP/RADTRAD code was used in analyzing rod ejection accidents (REA) and fuel handling accidents (FHA) [26]. This code uses mathematical models to calculate the EAB, LPZ, and CR using the following formula:

For doses at the EAB and LPZ:

(1) DOSECEDE=Release ×XQ×Brrate×InhaDCFremCEDE/Ci inhaled

(2) DOSEEDE=Release ×XQ× Submersion DCF 

(3) $${\mathrm{DOSE}}_{TEDE}=DOSE_{CEDE}+DOSE_{EDE}$$

Calculations for Control Room Doses [24]:

(4) $${\mathrm{DOSE}}_{\mathrm{CEDE}}={\mathrm{Time}}_{CR\_conc}\times {\mathrm{Time}\;}_{increament\;duration}\times {\;\mathrm{Br}}_{rate}\times {\mathrm{Inha}}_{DCF}\times {\mathrm{Occ}}_{factor}\;$$

(5) $${\mathrm{DOSE}}_{\mathrm{EDE}}={\mathrm{Time}}_{CR\_conc}\times {\mathrm{Time}\;}_{increament\;duration}\times {\mathrm{Submersion}}_{DCF}\times {\mathrm{Inha}}_{DCF}\times {\mathrm{Occ}}_{factor}$$

(6) $${\mathrm{DOSE}}_{\mathrm{TEDE}}={\mathrm{DOSE}}_{\mathrm{CEDE}}+{\mathrm{DOSE}}_{\mathrm{EDE}}$$

Thyroid and whole-body doses are estimated based on the curies of iodine and noble gases released into the environment using the mathematical formula below:

Thyroid doses at the EAB and LPZ:

(7) Dthy=DOSECEDEXQEAB×BR×∑i(Qi×DCFi,thy) 

Whole-body doses at EAB and LPZ:

(8) Dwb=XQ×LPZ×∑i(Qi×DCFi,thy) 

where:

*(X/Q)_LPZ_*: ADF at *LPZ*, s/m^3^;
*wb*: whole body;
*X/Q*: atmospheric dispersion;
*(X/Q)_EAB_*: Atmospheric Dispersion Factor (ADF) at EAB, s/m^3^;
*B_R_*: Breathing rate, m^3^/s;
*Q*: release activity, Ci;
*DCF*: Decontamination Factor (rem/Ci);
*i*: isotope;
*thy*: thyroid;
*D*: Dose;

Calculations for Control Room Dose:

(9) $$D_{\mathrm{CR},\mathrm{thy},i\;}=Q_{CR,i}\times \mathrm{BR}\times {\mathrm{DCF}}_{i}$$

where:

BR = (3.47 × 10 m^3^/s)

The concentration of isotope i at the control room intake is given by:

(10) $$Q_{\mathrm{intake}\;}=X/Q_{CR}\times R_{i}\mathrm{Page}:\;5$$

where:

($X/Q_{CR}$) = control room atmospheric diffusion factor;
R*_i_* = release rate of radionuclide *i* from the pool;

Control Room Whole Body Dose:

(11) $$D_{\mathrm{CR}-\mathrm{wB}\;}=\left({\mathrm{DCF}}_{\mathrm{gamma}}/\mathrm{GF}\right)\times A_{\mathrm{CR}}\times \left(Occ/V_{\mathrm{CR}}\right)\times \sigma t\;\mathrm{Page}:\;5$$

where:

GF = 1173/V^0.338^;
*Occ* = Control Room Occupancy;
V_CR_ = Volume of Control Room;
A_CR_ = Average Activity in Control Room;

(12) $$D_{\mathrm{CR}-\mathrm{skin}\;}=\left({\mathrm{DCF}}_{\mathrm{gamma}}/\mathrm{GF}+{\mathrm{DCF}}_{beta}\right)\times A_{\mathrm{CR}}\times \left(Occ/V_{\mathrm{CR}}\right)\times \sigma t$$

(13) DEAB−WB =XQ×DCFgamma×Curies released

(14)  DEAB−skin =XQ×DCFbeta×Curies released

This study used these models to estimate the EAB, LPZ, and CR doses in the case of a postulated REA and FHA, as described below. The following assumptions were used for FHA and REA modeling in RADTRAD. Non-iodine halogens like bromine are not included in the source terms due to their short half-lives and low activity concentration after 72 h, especially for calculating LPZ and CR doses. Although some of these half-lives existed within two hours, making them critical in the calculation for exclusion area boundary, their concentrations were almost negligible. As a rule of thumb, the accident duration is assumed to last 720 h (30 days). The fission inventory products are also assumed to be released based on the maximum power level, which is the product of thermal power and 1.02. This corresponds to current fuel enrichment and fuel burnup. In the case of APR1400, its thermal power will be (3983 × 1.02 = 4062.66 MWth). The DCD analysis shows containment of iodine chemical form of 95% cesium iodide, 4.85% elemental iodine, and 0.15% organic iodide. The fuel damage fraction is assumed to be 0.004 across all reactors. Maximum release to the environment is within 2 h, representing the worst 2-h doses at the exclusion area boundary. In addition, the control room dose analysis assumed the worst-case scenario; therefore, no filters were incorporated into the calculations. In this analysis, there was no reduction or mitigation of noble gas radionuclides released from the primary system. The FHA and REA analysis assumed worst-case atmospheric dispersion X/Q factors for conservativeness.

#### 2.1.1. Rod Ejection Accident Model

A control rod is ejected, resulting in a quick insertion of positive reactivity. A cladding breach or melt failure typically destroys a fuel rod section. As a result, the containment atmosphere is instantly and uniformly mixed with all the activity released from the fuel. Consequently, primary coolant leaks at the maximum rate allowed by design basis restrictions into both steam generators. The leaking will continue until the reactor is depressurized and the primary and secondary systems are at the same pressure. Then, the condenser releases activity into the environment until isolation is achieved [27]. Table 2 specifies REA analysis parameters.

#### 2.1.2. Fuel Handling Accident Model

In the hypothetical fuel handling accident (FHA), a fuel assembly is projected to be dumped and damaged during fuel handling. The accident can occur in the containment or the spent fuel pool (SFP) located in the auxiliary building’s (AB) fuel handling area. If radioactive material escapes the spent fuel pool or containment building, the leakage of radioactive material from the fuel-handling area emergency ventilation system is speculated to be discharged into the environment. The analysis setup is such that the inputs and assumptions apply to FHA occurring in both SFP and containment [23]. Parameters for FHA are shown in Table 3 below.

### 2.2. RASCAL Model on Long-Term Station Blackout

The Source Term to Dose (STDose) model embedded in RASCAL estimates projected radiation doses from the plume downwind during a severe accident. First, the model produces a time-dependent source term with a graph depicting the rate at which each radionuclide is released from the facility. Next, the time-dependent release rate is input into the atmospheric dispersion model. Next, due to deposition, the atmospheric dispersion and transport model calculates radioactive concentrations in the air and ground. Following that, the calculated concentrations are used to determine anticipated dosages. Cloudshine and inhalation from the plume and ground shine from deposited radionuclides are the dosage pathways (assuming four days of exposure to the ground). The RASCAL code recommends a 15-min interval of weather information for 72 h to make an adequate prediction.

#### The Long-Term Station Blackout Accident

The phrase “station blackout” is defined by the United States Nuclear Regulatory Commission’s 10 CFR 50.2 as the total loss of alternating current electric power to a nuclear power plant’s essential and non-essential switchgear buses [28,29]. The loss of offsite power co-occurs with turbine trip and failure of the onsite emergency AC power system, but not the loss of available AC power to buses fed by station batteries via inverters or electricity from “alternative AC sources.” [29]. The repercussions of a station blackout could be severe since numerous safety systems required for reactor core decay and containment heat removal rely predominantly on AC power. Furthermore, supposing the incident is prolonged, also known as a Long-Term Station Blackout, there will be a cooling loss in the reactor core due to increased temperature. The outcome will be significant core damage and subsequent escape of radioactive source terms outside the containment resulting in the offsite release.

Employing the same methodology for the Long-Term Station Blackout at the Arkansas Unit 1 power plant provided in the workbook, the methodology was tailored to suit the location in Ghana and specific reactors’ parameters derived from the literature. Table 4 displays relevant parameters used in simulating hypothetical LTSBO in Ghana.

## 3. Results

### 3.1. The RADTRAD Analysis Results

The SNAP/RADTRAD code evaluated the TEDE of the exclusion area boundary, the low population zone, and the control room doses. In addition, it further displays the contribution of the whole-body and thyroid doses to the TEDE of the EAB, LPZ, and control room. The RADTRAD output was verified by comparing the results with the FHA analysis published in the Design Control Document Tier 2 Chapter 15 [23].

#### 3.1.1. The Analysis of Rod Ejection Accident

**Figure 1:**
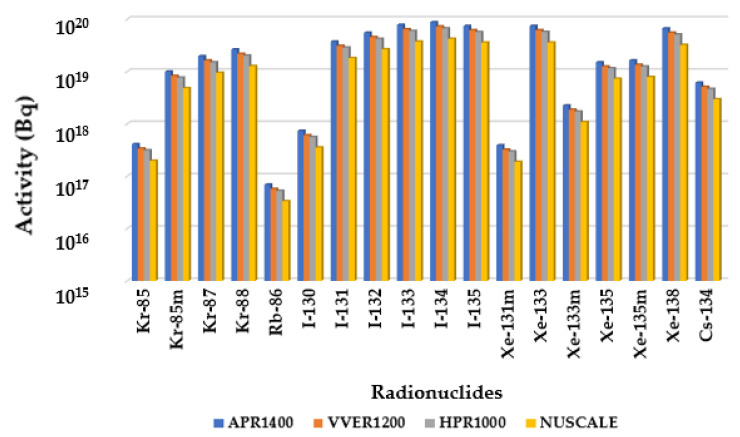
Comparison of rod ejection accident source term activity inventory in becquerels.

Figure 1 compares the radionuclide activity among APR1400, VVER1200, HPR1000, and Nuscale Power Module. It shows that the reactor’s power is directly proportional to the activity of each source term. Activity decreased with reducing power across the reactors in the following order APR1400 < VVER1200 < HPR1000 < Nuscale Power Module. Additionally, the activity levels of the radionuclides released by various reactors followed the same trend, in that I-134 was the highest among all four reactors, followed by I-133 and the rest. This was in the descending order of the reactors’ powers; APR1400, VVER, HPR1000, and Nuscale Power Module. In no case did the activity of a radionuclide in one reactor deviate from the trend.

**Figure 2:**
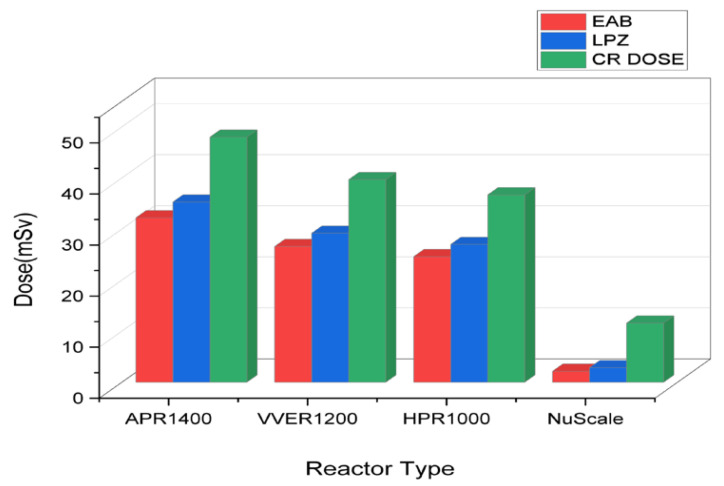
REA TEDE comparison of exclusion area boundary, low population zone, and control room.

Comparing the total effective dose equivalents TEDEs in Figure 2, the control room showed the highest doses, followed by the low population zone and exclusion area boundary. No filters were used to analyze the control room doses to ensure conservativeness. The reason doses from the control room were the highest was because of their proximity to the radiation source. Hence, in this accident, the closer the distance, the higher the dosage. In addition, the TEDE decreased in descending order of reactor power, APR1400 < VVER1200 < HPR1000 < Nuscale Power Module. During the rod ejection accident, power plays a critical role in the amount of doses released. This is because there is an abrupt increase in positive reactivity when the rod is ejected. The heat produced during reactor operation is proportional to the reactor’s power, affecting the fuel melt or breach proportion. Therefore, for a single rod ejected from reactors with different thermal powers, the fraction of breach or melt will depend on the reactors’ power level.

**Figure 3:**
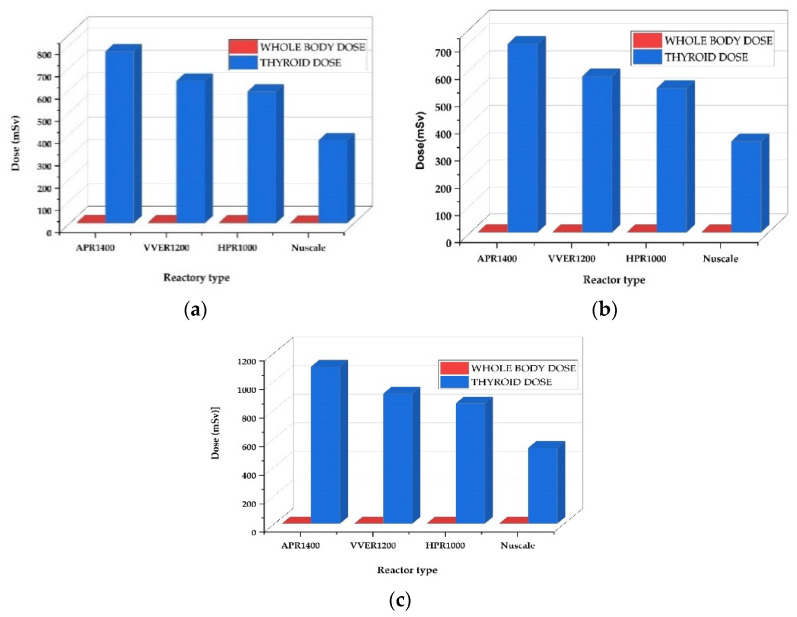
REA thyroid and whole-body dose comparison at the EAB, LPZ, and CR during fuel handling accidents. (**a**) represents doses at the exclusion area boundary. (**b**) represents doses at the low population zone. (**c**) represents doses in the control room.

Figure 3 displays a high spike in thyroid doses with an almost insignificant whole-body dose. However, thyroid doses are directly proportional to the reactors’ power in the following order: APR1400 < VVER1200 < HPR1000 < Nuscale Power Module. When calculating thyroid and whole-body dosages, the contribution of iodine and noble gases released into the atmosphere is considered. The source terms emitted by the iodine source were higher than those of the noble gases. Due to the thyroid organ’s higher radiosensitivity than the skin, little iodine raises thyroid dosages in dose calculations. Furthermore, most noble gases in whole-body doses have shorter half-lives and lower activities than iodine.

#### 3.1.2. Analysis of Fuel Handling Accident

**Figure 4:**
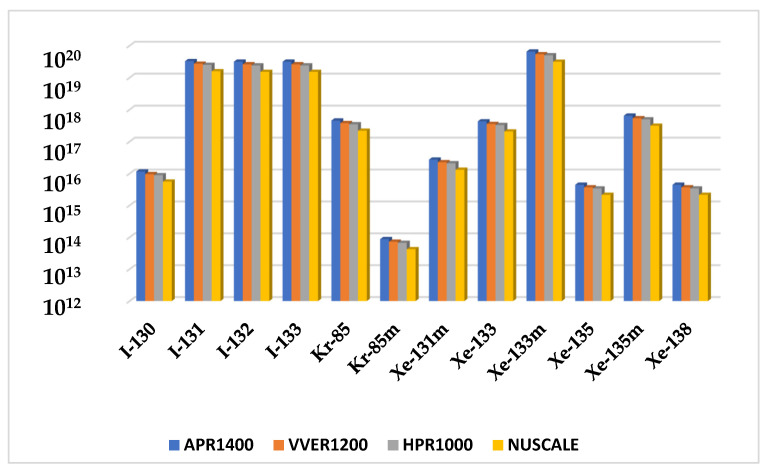
Comparison of fuel handling accident source term activity inventory.

Activities of the radionuclide inventory for fuel handling accidents were compared. APR1400 showed the highest activity among the source terms, followed by VVER1200, HPR1000, and Nuscale. Due to the extremely low activity of some of the source terms, the logarithmic scale graph was used to show the existence of the radionuclides whose activities were insignificant to the others. The graphical description is shown in Figure 4. Before replacing old fuel with fresh fuels, the reactor is shut down, borated, and cooled to ensure perfect refueling conditions. Therefore, during a fuel handling accident, the reactor’s power plays a minimum role in the source terms released. However, the fraction of damaged fuel is what directly affects the source terms released. The results below show that the activity levels of source terms are very close even though the reactor has significant thermal power differences.

**Figure 5:**
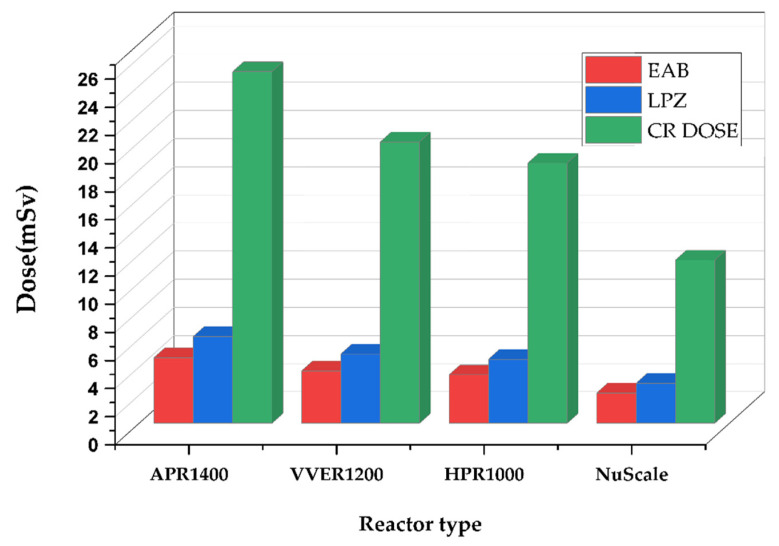
FHA TEDE comparison of exclusion area boundary, low population zone, and main control room.

Figure 5 displays the total effective dose equivalent (TEDE) for the exclusion area boundary, low population zone, and control room. Again, the control room had the highest doses, followed by the low population zone and exclusion area boundary. The magnitude of doses received results from the source term activities. Since the fraction of fuel damage compared to the total fuel assemblies was 0.004 for all four reactors, it is assumed that the doses released from the containment will correspond with the contribution of source terms released. In addition, the exclusion area boundary is closer to the power plant than the low population zone, so it is expected to have a higher dosage. On the contrary, doses for low population zones tend to be higher, possibly resulting from elevated release caused by unstable climatic conditions such as wind speed and wind direction. Finally, the control room had the highest doses because it was the closest to the fuel source and accident location. In verifying the correctness of this analysis, results for APR1400 displayed in the design control documentation chapter 15 are compared in Table 5 below.

**Figure 6:**
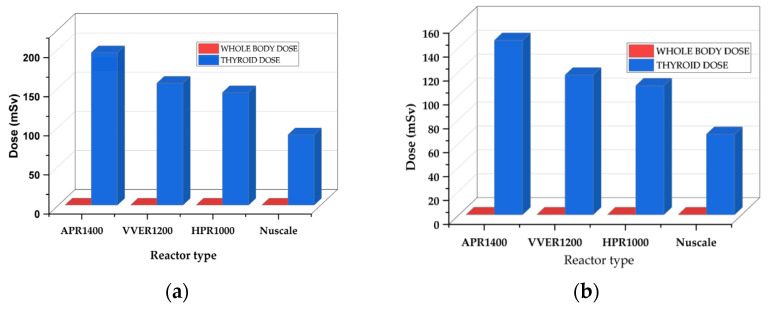

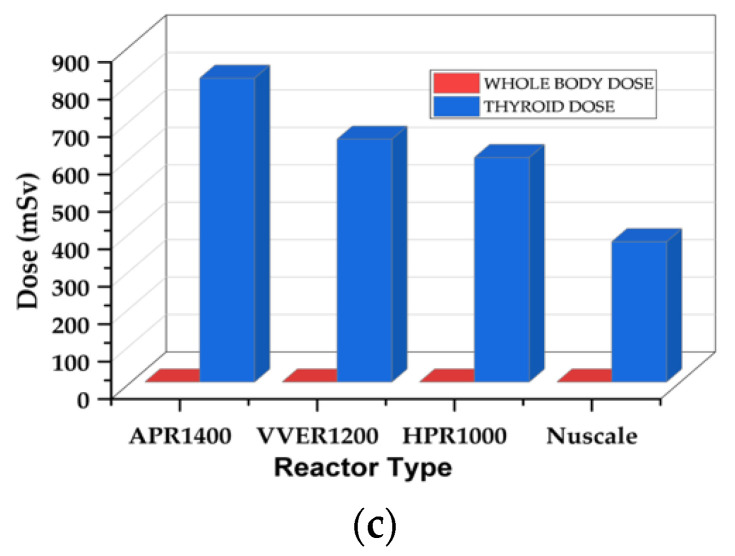
FHA whole-body and thyroid doses at the Exclusion Area Boundary, Low Population Zone, and Control Room Doses during fuel handling accidents. (**a**) represents doses at the exclusion area boundary. (**b**) represents doses at the low population zone. (**c**) represents doses in the control room.

The thyroid doses for EAB, LPZ, and MCR increased with the reactor’s increasing power with insignificant whole-body doses in the order; APR1400 < VVER1200 < HPR1000 < Nuscale Power Module. Thyroid doses were high across all reactors in the control room, proceeded by the exclusion area boundary and low population zones, as shown in Figure 6. The thyroid and whole-body doses are calculated based on the contribution of iodine and noble gases released into the environment. The iodine source terms released were higher than the noble gases. In dose calculation, small iodine elevates thyroid doses due to the high radiosensitivity of the thyroid organ compared to the skin. Additionally, most noble gases contributing to whole-body doses had very short half-lives and lower activities.

### 3.2. The RASCAL Results

**Figure 7:**
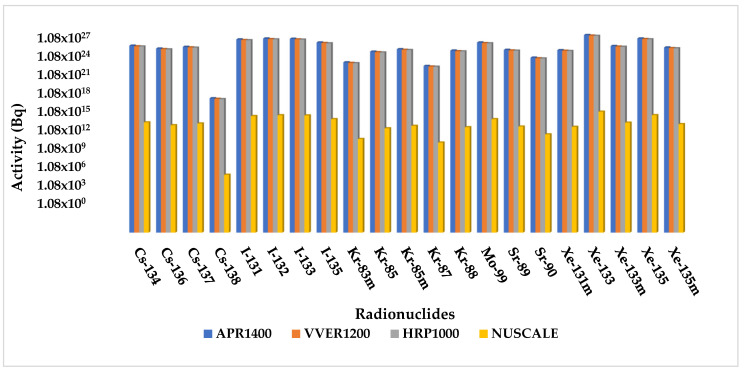
Radionuclide source term comparison for APR100, VVER1200, HPR1000, and Nuscale during Long-term Station Blackout Accident.

Figure 7 evaluated source term activities for long-term station blackout accidents. APR1400 showed the highest actives among the source terms, followed by VVER1200, HPR1000, and Nuscale. The concurrent loss of offsite power and turbine trip may disrupt heat removal in the reactor core, causing core damage and release of radionuclides into the environment. The amount of power required by each reactor to facilitate core cooling corresponds with the specific reactor’s thermal capacity. Therefore, the impact of the core meltdown is proportional to the number of damaged fuel assemblies. Therefore, the consequence of a long-term station blackout is affected by both power and the number of damaged fuels.

**Figure 8:**
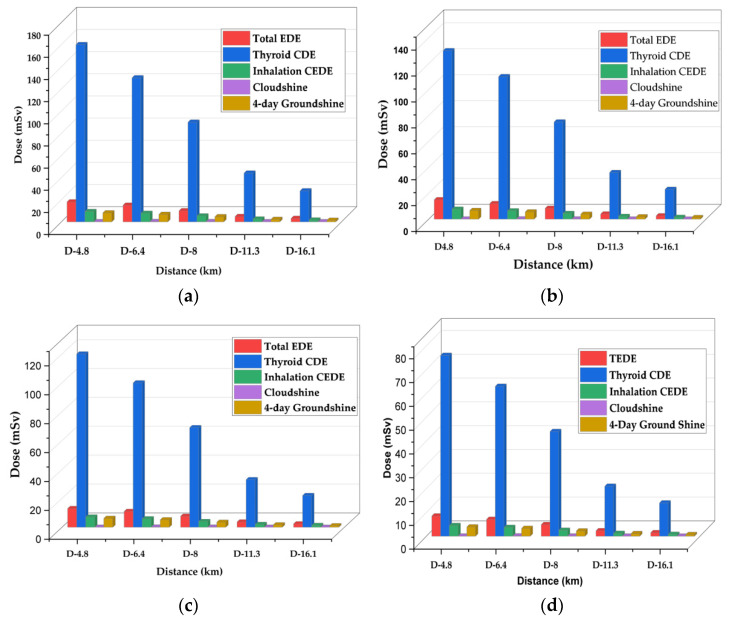
Graph of maximum TEDE, Inhalation CEDE, Thyroid, Cloudshine, and 4-day ground shine during long-term station blackout. (**a**) represents maximum doses within a 16 km radius from the APR1400 reactor. (**b**) represents maximum doses with a 16 km radius from the VVERR reactor. (**c**) represents maximum doses with a 16 km radius from the HPR1000 reactor. (**d**) represents maximum doses within a 16 km radius from the Nuscale Power Module.

The maximum doses received at vantage points within 16 km during long-term station blackout were assessed for all four reactors under consideration for this study. Again, Figure 8 displays decreased doses with the reactors’ decreasing power in the following order, APR1400 < VVER1200 < HPR1000 < Nuscale Power Module. As already established, doses at a distance are influenced mainly by the contribution of radionuclide source terms released during the accident. For example, thyroid doses affected by the level of iodine are highest because the iodine contents are high; notwithstanding this, even small iodine contents generally spike thyroid doses.

**Figure 9:**
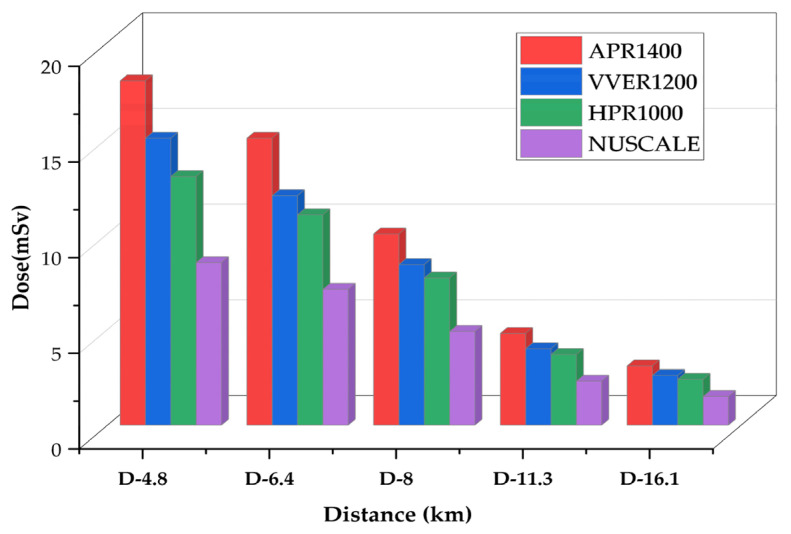
Graph of TEDE comparison during long-term station blackout within 16 km for APR1400, VVER1200, HPR1000, and Nuscale Power Module.

Although TEDE is seen in Figure 8, Figure 9 gives a magnified representation of the TEDE among the reactors. The trend in the TEDE at 16 km distance follows the order of APR1400, VVER1200, HPR1000, and the Nuscale Power Module. As usual, the dose level is consistent with the activities of released radionuclides. Doses are the contributions of various radionuclides released and present within a specific time.

## 4. Discussion

Emergency planning zone is one of the critical considerations in NPP deployment, which evidence-based studies and safety calculations must follow. The discussion for this research is focused on the fulfillment of the objectives of the study stated in chapter 2, where EAB, LPZ, and Main Control Room doses for APR1400, VVER1200, HPR1000, and Nuscale Power Module were estimated using the RADTRAD code. Additionally, the long-term station blackout simulated with RASCAL on all the reactors under study will be discussed. Finally, factors influencing the outcomes will be stated with regulatory standards in perspective.

### 4.1. The Analysis of Rod Ejection and Fuel Handling Using the SNAP/RADTRAD Code

In the Rod Ejection Accident analysis, as shown in Figure 1, the radionuclide source term inventories were compared. A general trend in the radionuclide activities was seen in the different reactors under study. Radionuclide activities were highest for APR1400, followed by VVER1200, HPR1000, and Nuscale Power Module. The reactor power is the topmost contributor to radionuclide release besides the atmospheric dispersion and fraction of breached/melted fuels. Other possible factors could have been the fraction of breach, the fraction of clad melt, and the radial peaking factor, which were kept constant for all the reactors and therefore showed no impact.

The Total Effective Dose Equivalent (TEDE) analysis for EAB, LPZ, and CR doses displayed in Figure 4 also follows a trend where TEDE decreases with decreasing power of the reactor in the order APR1400 < VVER1200 < HPR1000 < Nuscale Power Module. Again, power is essential in the doses of radionuclide doses released. Additionally, it can be argued that activity is directly proportional to the doses received. The thyroid and whole-body dose calculations for EAB, LPZ, and CR represented in Figure 3 also showed the same trend in decreasing order of APR1400, VVER1200, HPR1000, and Nuscale Power Module. Thyroid doses were highest in both EPZs and CR, with insignificant whole-body doses. Due to the influence of reactor thermal power on the accident progression of rod ejection accident, the magnitude of doses released correlated with the powers of the various reactors.

Like the rod ejection accident, as shown in Figure 4, the fuel handling accident had activities increasing with the reactor’s increasing power and number of fuel assemblies. However, their magnitudes were close to each other. The most limiting parameter affecting fuel handling accidents is the fraction of damaged rods. The number of fuels damaged during the accident is critical in determining the amount of radioactive release to the environment. The fraction of damage used in the calculation was kept constant at 0.004 across the reactors, whiles the number of fuel assemblies varied for each reactor. Hence, the code automatically calculated the number of damaged fuels. Because FHA has little to do with power, source term analysis showed close magnitudes among the doses received from the reactors, even though the difference between the powers was significant. TEDE calculations for the fuel handling accidents (FHA) decreased in the following order APR1400, VVER1200, HPR1000, and Nuscale Power Module shown in Figure 5. As stated above, FHA is significantly affected by the total number of fuels and the fraction of damaged fuels. The control room had the highest doses, followed by a low population zone and exclusion area boundary.

Comparing the doses from REA and FHA, it can be noticed that the scale for REA is ten times higher for all graphs compared to FHA. Due to the small fraction of 0.004 of damaged fuel used in the calculation for FHA, the doses released are lower than the doses for REA. Since FHA is predominantly affected by the fraction of damaged fuel, doses do not reflect the magnitudes of reactors’ power. On the other hand, doses estimated for REA directly reflect the reactors’ power. The REA and FHA showed a significant difference between the thyroid dose represented at the exclusion area boundary, low population zone, and control room. The whole-body doses were almost insignificant, fulfilling the requirement of the 1O CFR 100.11. The 1O CFR 100.11 states that whole body and thyroid doses at the exclusion area and low population zone should be within the limit, whereas “well within” means that the whole-body dose should be less than 25% of the thyroid dose. International regulations propose that doses to an individual in the control room within 72 h of a postulated design basis accident should not exceed 50 mSv, according to the General Design Criteria 19 (GDC 19) [30]. In addition, control room calculations for rod ejection and fuel handling accidents also fulfilled the Standard Review Plan (SRP) 6.4 and the 1O CFR 100.11 criteria for control room dose limit as 50 mSv whole-body and 3000 mSv thyroid dose [31].

### 4.2. The Analysis of Long-Term Station Blackout Using the RASCAL Code

Using the RASCAL Code, the Long-Term Station Blackout (LSBO) consequence analysis estimated on APR1400, VVER1200, HPR1000, and Nuscale Power Module generated sources term activities in decreasing order of reactor power; APR1400 < VVER1200 < HPR1000 < Nuscale Power Module. Since reactor core decay and heat removal largely depend on the availability of AC power which supports critical safety systems, the consequence of LTSBO will be directly proportional to the reactor’s power and the number of fuel assemblies. Analysis of the maximum doses showed that APR1400 had the highest doses, followed by VVER1200, HPR1000, and Nuscale Power Module. Doses reduced with increasing distances in all the reactors. For example, at a distance of 4.8 km from the power plant, the TEDE produced by APR1400 was 18 mSv, while VVER produced 15 mSv, HPR1000 produced 13 mSv, and Nuscale produced 8 mSv within 72 h. At 16 km, which the guidelines consider a safe zone, APR1400 produced a TEDE of 3.1 mSv, VVER1200 had 2.6 mSv, HPR1000 had 2.4 mSv, and Nuscale Power Module gave 1.50 mSv. As recommended by the ICRP and the NRC, the dose limits according to 10 CFR 20, total effective dose equivalent exposure to members of the public should not exceed 1 mSv (0.1 rem) per year [18]. Based on the results, an individual member of the public situated at a 16 km distance away from the power station may likely exceed the annual dose limit. However, assuming the result represents collective doses of many individuals, then individual doses at that distance may be negligible and, therefore, safe within the 16 km periphery.

## 5. Conclusions

The study on emergency planning zone and control room habitability sought to assess doses received at the exclusion area boundary and control room in case of postulated rod ejection and fuel handling accidents using the SNAP/RADTRAD code. The results above indicate that the highest doses will be found in the control room, although they will be within the regulatory limit of 50 mSv. At the exclusion area boundary, the low population zone and control room doses were reduced in descending order of reactor power and number of fuel assemblies. That is, APR1400 < VVER1200 < HPR1000 < Nuscale Power Module. The RASCAL code estimated the radiological consequence on the members of the public in case of a severe accident. The analysis focused on maximum doses released at the recommended 16 km safe zone, assuming a long-term station blackout lasted for 72 h. In addition, distance and time reduce doses, validating that spatial and temporal factors play a role in radiation dosimetry. Again, maximum doses for thyroid, inhalation, cloud shine, and ground shine were reduced in the order of reactor power; APR1400 < VVER1200 < HPR1000 < Nuscale Power Module. In terms of ALARA planning, the Nuscale Power Module proved that offsite doses were as low as reasonably achievable. However, aside from the emergency planning zone, Ghana must consider other safety, security, and economic factors before finalizing a reactor type for deployment.

## Figures and Tables

**Table 1:** Nuclear power plants specific parameters for RADTRAD and RASCAL codes.

Parameters	HPR 1000	VVER 1200	APR 1400	NUSCALE
Containment volume	~73,500 m^3^/2,595,628 ft^3^	2917.82 m^3^/10,3041.8409 ft^3^	9000 m^3^/3,178,320 ft^3^	1791.428571 ft^3^
Control room volume	Assumed 5663 m^3^ (200,000 ft^3^)	Assumed 5663 m^3^ (200,000 ft^3^)	5663 m^3^ (200,000 ft^3^)	Assumed 5663 m^3^ (200,000 ft^3^)
Core Thermal Power	3050 MWth	3200–3300 MWth	3983 MWth	1920 MWth
Fuel Assemblies	117 (264 fuels in each assembly)	163 assemblies (317 fuels in each)	241 assemblies (236 rods in each assembly)	17 × 17 (264)
Fraction of Damage for FHA	4.0 × 10^−3^	4.0 × 10^−3^	4.0 × 10^−3^	4.0 × 10^−3^
Fraction of clad breach for REA	0.1	0.1	0.1	0.1
Fraction of breach > melt for REA	2.5 × 10^−3^	2.5 × 10^−3^	2.5 × 10^−3^	2.5 × 10^−3^
Radial Peaking factor	1.8	1.8	1.8	1.8
Type of Reactor	PWR	PWR	PWR	PWR

**Table 2:** Rod ejection accident analysis parameters using RADTRAD.

Parameter	Value
Fraction of clad breached	Calculated from the fraction of breach and number of fuel assemblies
Fraction of breach > melt	2.5 × 10^−3^
Noble Gas	Gap 0.1; melt 1.0
Iodine	Gap 0.1; melt 0.5
Alkali metals	Gap 0.12; melt 0.5
Gap release duration	1 × 10^−4^ h.

**Table 3:** Fuel handling accident analysis parameters using RADTRAD.

Parameter	Value
Number of Assemblies damaged	Calculated from the fraction of breach and number of fuel assemblies
Fraction of fuel damaged	4.0 × 10^−3^
I-131	0.08
Kr-85	0.1
Other NG	0.05
Other Iodine	0.05
Alkali metals	0.12
Gap release duration	2 h
Containment Leak Rate to Environment	1.0 × 10^12^%/day for the duration of the accident

**Table 4:** Long-term station blackout analysis parameter using RASCAL.

Parameter	Value
Event type	Nuclear Power Plant
City, Country	Ghana, Tema
Time zone	Greenwich Meridian Time (GMT)
Lat/Long/Elevation	5.0000° N, 0.0000° E, 14 m
Containment type	PWR Dry Ambient
Leak rate	2%/d
Spray	off

**Table 5:** Comparison of FHA Analysis using verified DCD results [23].

TEDE	EAB	LPZ	CR DOSE
**APR1400**	**4.66 × 10^0^**	**6.17 × 10^0^**	**2.50 × 10^1^**
VVER1200	3.72 × 10^0^	4.93 × 10^0^	2.00 × 10^1^
HPR1000	3.44 × 10^0^	4.55 × 10^0^	1.85 × 10^1^
NuScale	2.15 × 10^0^	2.84 × 10^0^	1.16 × 10^1^
**DCD for APR1400**	**6.30 × 10^1^**	**6.30 × 10^1^**	**6.25 × 10^0^**
**Allowable TEDE limit**	**5.00 × 10^1^**	**6.30 × 10^1^**	**6.30 × 10^1^**

## Data Availability

The data supporting this study’s findings are available on request from the corresponding author.
